# Life expectancy and health care spending in South Asia: An econometric analysis

**DOI:** 10.1371/journal.pone.0310153

**Published:** 2024-12-23

**Authors:** Bharat Ram Dhungana, Jitendra Kumar Singh, Samrat Dhungana

**Affiliations:** 1 School of Business, Pokhara University, Pokhara, Gandaki Province, Nepal; 2 Department of Community Medicine, Janaki Medical College, Tribhuvan University, Janakpur, Madhesh Province, Nepal; 3 Faculty of Health Sciences, Rajarshi Janak University, Janakpur, Madhesh Province, Nepal; 4 Faculty of Arts and Sciences, Harvard University, Boston, Massachusetts, United States of America; University of Foggia, ITALY

## Abstract

Affordable health care is often a result of increased government spending on the health sector. Out-of-pocket expenses remain the primary health care funding source in many South Asian nations. Lack of adequate public funding for health services, difficulty in obtaining health insurance, and high out-of-pocket costs can result in indebtedness, reductions in actual consumption, and decreased access to health care services. The study examines life expectancy and health care spending in South Asian countries. The life expectancy of South Asian countries is studied as a health outcome in relation to health care spending, gross national income per capita, and expected years of schooling. This study is based on secondary data from World Bank statistics that covers eight South Asian countries from 2000 to 2021, for a total of 176 pooled time series and cross-sectional observations. The data were analysed using econometric models, including the cross sectional dependency test, panel unit root test, panel co-integration test, vector error correction model, pair-wise Granger causality test, and Wald test statistics. The vector error correction model results indicate that health care spending, anticipated years of schooling, and gross national income per capita have a long-run association with health outcomes. Health care spending, per capita gross national income, and expected years of education have all greatly enhanced life expectancy in South Asian countries. An effective health strategy is necessary to increase people’s healthy life expectancy and well-being. To accomplish this, government may need to promote school enrolment to help people become more health literate and aware of their health outcomes. As a result, persons with more years of schooling have better health, higher levels of well-being, healthier habits, and ultimately, a longer life expectancy. This study will provide an important insight to policymakers in improving health outcomes through targeted and sustained health care spending in South Asia.

## Introduction

Globally, the government increasingly prioritizes ensuring good health and well-being of the people [1,2]. However, in low income nations, investing in the health sector often have a major financing constraint and limited impact on health outcomes. The health spending capacity of a nation significantly influences health outcomes [3]. Several evidence shows that there is a nexus between health care spending and health outcomes and it acts as a critical measure of healthcare policies effectiveness [4–6]. Yet, the question of improving health outcomes is a significant health policy issue in low income nations. Realizing the significance of good health policy for better health outcomes, researchers conducted this study to explore the nexus between life expectancy and health care spending in South Asia.

South Asia is a home of almost a quarter of the world population struggling for in accessing quality health care and outcomes [7]. This region includes eight nations—Afghanistan, Bangladesh, Bhutan, India, Maldives, Nepal, Pakistan, and Sri Lanka. Health care spending remains low, relying heavily on out-of-pocket costs and limited public financing [8]. This region is facing economic resource constraints, gaps in quality of health care, dominant of costly private health delivering institutions, and inadequate health professionals [9]. A critical challenge of poverty, quality education and health services complicate the efforts of government to improve health outcomes [10]. Addressing these issues requires increased investment in health care, effective policy reforms, and the use of new technologies to ensure equitable access to quality health services.

Life expectancy is widely used measurement indicator of health outcome. A population health indicator that considers both length and quality of life is healthy life expectancy (HLE). It is all part of highlighting health disparities, focusing resources on health promotion, assessing the results of health policies, and planning for health, social, and fiscal policy [11]. Health is a crucial factor for productivity and economic growth. Due to its effects on population, participation, and production, good health can over time result in increased GDP (gross domestic product) (GDP) per capita [12]. Good health care system ensures to achieve good health and healthy life expectancy [6]. A major health policy issue is whether improvements in quality of life are consistent with increases in life expectancy.

The Grossman health capital model focuses on the issues related to health, health care demand, occupational choice, health preventions, and socio-economic inequalities in health [13]. This theory suggests that investing in health care spending ensures to improve life expectancy and health outcomes [14]. Adam Wagstaff argues that health care spending can improve health outcomes, but how this improvement will happen depends on the efficiency and adequacy of the health care system [15]. The theory developed by Samuel Preston explains that health care spending and income per capita significantly affect life expectancy [16]. Likewise, human capital theory developed by Gary Becker explains that investment in health and education improves the productivity and economic value of the people. The investment in health care improves human capital that facilitates to increase life expectancy and health outcomes [17]. These insights are important for policymakers aiming to improve health outcomes through targeted and sustained health care spending in South Asia.

Several studies found a positive relationship between health care expenditure and health outcomes [18,19]. The primary influence of health care spending determines a country’s level of wealth. Health care expenditure is mainly influenced by income [20,21]. However, a high health care expenditure sometimes could signify poor health because of epidemics, natural disasters, aging populations, greater demand for medical interventions, and inadequate preventive measures [22]. Effective policymaking at the national and regional levels requires understanding overall health care expenditures as a share of the total gross domestic product (GDP), including public and private health care expenditures [23]. Poor health outcomes and ineffective use of health care resources are linked to low health literacy [24]. Health literacy has become increasingly concern for improving health outcomes in many developing countries [25].

The prominence of societal issues today has led to a rising understanding that achieving inclusive growth and sustainable development depends critically on the health and well-being of a population [26]. In this sense, the correlation between life expectancy and health care spending has drawn much attention as a crucial determinant of a population’s health results [27]. Infrastructure, accessibility, and quality issues in health care present special concerns for South Asia [28,29]. Increased health care spending can significantly improve life expectancy by expanding health services and preventive measures. However, differences in health expenditure and resource allocation often result in differential improvements in health outcomes [4]. Thus, understanding the dynamics of health expenditure and life expectancy in South Asia is critical for effective health care policy development.

Based on the above study, it is found that health care expenditure matters for the health outcomes. However, there is a limited study using econometric analysis to examine the relationship between health care expenditures and life expectancy in South Asia. This study aims to fill the research gap often overlooked the variables gross national income per capita and expected years of schooling in many studies. Moreover, theoretical contributions from Grossman’s health capital model, Adam Wagstaff’s efficiency model, Samuel Preston’s income-health nexus, and Gary Baker’s human capital theory, all of which are rarely used in research. Therefore, this study aims to examine health outcomes in terms of life expectancy with wide range of health care spending indicators—current health expenditures, domestic health expenditures, out-of-pocket payments, and private health expenditures including gross national income per capita, and expected years of schooling. It is anticipated that evidence-based programmes and efforts can be established by policymakers, healthcare providers, and relevant authorities to improve the general well-being and life expectancy of the people in South Asia.

## Materials and methods

The study intends to investigate South Asian nations’ health care spending and life expectancy, hence all South Asian nations were included. South Asia comprises eight countries: Afghanistan, Bangladesh, Bhutan, India, Maldives, Nepal, Pakistan, and Sri Lanka. This study is based on secondary sources of data that were acquired from World Bank Statistics and Global Health Expenditure Database [30,31]. The variables used in the study were life expectancy, health care expenditure (current health expenditures, domestic health expenditures, out-of-pocket payments, and private health expenditures), anticipated years of schooling, and gross national income per capita. It includes 22 years of data from 2000 to 2021, with a total of 176 pooled time series and cross-section observations from South Asian countries.

The econometric approach was used to investigate the life expectancy and health-care spending in South Asian countries. The panel data were analysed using econometric models, including cross-sectional dependency test, panel unit root test, panel cointegration test, vector error correction model, pair-wise Granger causality test, and Wald test statistics. First, we used the Pasaran CD test to investigate cross-sectional dependence. Then we performed a panel unit root test to ensure that the first and second differences were stationary. Again, we used the panel cointegration test to figure out if there is a long-term relationship between the panel data. After determining the variables’ cointegration, we employed the VECM technique to estimate the long-run adjustment process. We also utilised the Granger causality test and Wald test statistics to assess the causal relationships and short-run cumulative effects of the independent factors on the dependent variable. Finally, we ran a diagnostic residual test to validate the model. We utilised life expectancy at birth to assess population health outcomes [32]. Table 1 provides the name, abbreviation, and description for each variable.

**Table 1:** Variables and descriptions.

Variable Names	Acronyms	Description
**Dependent Variable** Life expectancy **Independent Variables** Current health expenditure Domestic health expenditure Out-of-pocket health expenditure Private health expenditure Gross national income per capita Expected years of schooling	LE CHE DOM OOPS PVTD GNIPC EYS	Life expectancy of people. It is a dependent variable. Current health expenditure as % of gross domestic product. Domestic health expenditure as % of current health expenditure Out-of-pocket (OOPS) as % of current health expenditure (CHE) Private health expenditure (PVT-D) as % current health expenditure (CHE) Gross national income per capita in USD measures the economic power of the people Expected years of schooling in years measure the education status of the people

## Results and discussion

### Cross sectional dependency

Panel-based unit root tests are powerful as compared to the unit root tests based on individual time series. Cross-sectional dependency in panel data refers to correlations or relationships among different entities (units) observed simultaneously across multiple time periods. It is, therefore, crucial first to ensure unbiased parameter estimates and valid inferences in panel data analysis [33]. Testing for cross-sectional dependence or contemporaneous correlation assesses interdependencies among units (individuals, firms, regions) simultaneously, crucial for accurate panel data modelling and interpretation [34]. By understanding these dependencies, researchers and policymakers can make more informed decisions based on robust econometric analyses. In this study, we employed the Pasaran CD test to examine cross-sectional dependence.

The test yielded a Pesaran’s test statistic of -0.675 (Pr = 0.4996) and an average absolute value of the off-diagonal elements of 0.232, indicating no significant cross-sectional dependence in the dataset (Table 2). Consequently, under the assumption of independent units suggested by the Pasaran CD test results, first-generation unit root tests such as the Augmented Dickey Fuller (ADF) or Phillips-Perron (PP) test can be appropriately employed. Thus, ADF test is applied to find the unit root test.

**Table 2:** Cross sectional dependency test.

R-squared:					Obs per group:			
Within	=	0.9060				min	=	18
Between	=	0.7318				avg	=	19.8
Overall	=	0.7538				max	=	20
corr(u_i,xb)	=	-0.5185				F(6,144)	=	231.32
						Prob > F	=	0.0000
LE		Coefficient	Std. err.	t	P>|t|	[95% conf. interval]		
EYS		1.083004	0.071394	15.17	0.000	0.9718888		1.22412
GNIPC		0.0006525	0.0000642	10.16	0.000	0.0005255		0.0007794
CHE		0.1759889	0.0828281	2.12	0.035	0.0122334		0.3397445
DOM		-0.0236023	0.0197896	-1.19	0.235	-0.062718		0.0155133
PVTD		-0.0158289	0.0278077	-0.57	0.570	-0.070793		0.0391351
OOPS		-0.0682574	0.0282124	-2.42	0.017	-0.124021		-0.0124935
_cons		58.84928	1.69126	34.80	0.000	55.50638		62.19218
sigma_u		3.0422511						
sigma_e		0.74502419						
rho		0.94342089						
F test that all u_i = 0: F (7, 144) = 88.11						Prob > F = 0.0000		
.xtcsd, pesaran abs								
Pesaran’s test of cross-sectional independence = -0.675, Pr = 0.4996								
Average absolute value of the off-diagonal elements = 0.232								

### Panel unit root test

Stationarity is crucial for time series and panel data [35]. A time series and panel data are stationary if its mean and variance are time-invariant. Time series econometrics often employs a stationary, linear, or nonlinear time series model after removing a trend from or taking a (log-) difference of a non-stationary economic time series [36]. Data from non-stationary time series should be differentiated into stationary data [37]. The panel unit root test summary has been presented in Table 3.

**Table 3:** Panel unit root test summary.

Variables	Level	First Difference	Second difference	Degree of Integration
LE	-1.36878	-0.69614	247.607	I (2)
	(0.0855)	(0.2432)	(<0.00001)	
CHE	20.6971	91.6434	------------	I (1)
	(0.1905)	(<0.00001)		
OOPS	14.9257	95.1899	------------	I (1)
	(0.5301)	(<0.00001)		
DOM	23.8893	101.199	------------	I (1)
	(0.0920)	(<0.00001)		
PVTD	15.1918	87.9291	------------	I (1)
	(0.5106)	(<0.00001)		
GNIPC	14.1163	79.0294	------------	I (1)
	(0.5901)	(<0.00001)		
EYS	23.2453	47.8819	------------	I (1)
	(0.1073)	(<0.00001)		

Table 3 shows that all the variables have unit roots or are non-stationary at a level I (0), where the P-value is inconsequential. Additionally, the table shows that the variable is stationary or has no unit root at the first and second differences, as shown by I (1) and I (2), respectively, as the P-value is significant at the first and second differences. The common unit root process includes Levin, Lin & Chu t*, and the individual unit root process includes Im, Pesaran, and Shin W-stat, ADF—Fisher Chi-square, and PP—Fisher Chi-square.

### Panel cointegration test

A cointegration test is utilized to determine whether there is a long-term association between different time series and panel data. Several non-stationary time series and panel data are tested for cointegrating relationships using the Johansen test [38]. The results of cointegration tests reveal situations in which two or more non-stationary time series and panel data are combined so that they cannot depart from equilibrium over the long term. Consider the generic equational model below:

$$\Delta y_{t}=\Pi y_{t-1}+\sum_{i=1}^{p-1}\Gamma_{i}\Delta y_{t-i}+u_{t}$$

The Panel Cointegration test among the health care spending variables and other variables is presented in Table 4. In this study, sample taken from 2000 to 2021with 176 observations including linear deterministic trend assumption with automatic lag length selection based on SIC with a max lag of 2.

**Table 4:** Panel cointegration test.

Kao Residual Cointegration Test						
Series: LE OOPS PVTD DOM GNIPC EYS						
Null Hypothesis: No cointegration		t-Statistic		Prob.		
ADF		-6.0838		0.0000		
Residual variance		0.5858				
HAC variance		0.2284				
Pedroni Residual Cointegration Test						
	With dimension			Between dimensions		
	Statistic	Prob.	Weighted Statistic	Prob.	Statistic	Prob.
Panel PP-Statistic	-8.1063	<0.00001	-4.9667	<0.00001	-6.7336	<0.00001
Panel ADF-Statistic	-6.0942	<0.00001	-4.6884	<0.00001	-5.1107	<0.00001

From Table 4, Kao and Pedroni Residual Cointegration test suggest that there is cointegration among life expectancy, out-of-pocket payments, private health expenditure, domestic health expenditure, gross national income per capita, and expected years of schooling at a 5% level. The Johnson Fisher Panel Cointegration test has been presented in Table 5. Here, sample taken from 2000 to 2021with 176 observations including linear deterministic trend assumption.

**Table 5:** Johnson fisher panel cointegration test.

Series: LE CHE OOPS GNIPC EYS				
Trend assumption: Linear deterministic trend				
Unrestricted Cointegration Rank Test (Trace and Maximum Eigenvalue)				
Hypothesized	Fisher Stat.		Fisher Stat.	
No. of CE(s)	(From trace test)	Prob.	(From max-eigen test)	Prob.
None	111.9	<0.00001	111.9	<0.00001
At most 1	286.1	<0.00001	173.9	<0.00001
At most 2	165.7	<0.00001	87.65	<0.00001
At most 3	96.02	<0.00001	71.00	<0.00001
At most 4	43.48	0.0001	38.44	0.0004
At most 5	24.27	0.0426	24.27	0.0426

Table 5 shows five cointegrating equations among the research variables at the 5% level based on trace statistics and the maximum Eigenvalue statistic. We proceed with the VECM approach to estimate the error correction coefficients since the test implies that cointegrated panel data contain an error-correction representation that reflects the long-run adjustment process.

### Vector error correction model (VECM)

This model is used to examine the long-term relationships between the variables. When the variables exhibit cointegration and are ordered one integrator, the VECM is employed. Since there is cointegration among all the variables, the VECM model (also called restricted VAR model) has been applied to find out whether there is a long-run association among the variables. The following equation has been developed to estimate VECM:

*Equation*: D(DDLE) = C(1)* (DDLE(-1) + 0.0233*DDOM(-1) + 0.0239*DOOPS(-1) - 0.0303* DPVTD(-1) - 0.0002*DGNIPC(-1) + 0.0172*DEYS(-1) + 0.0458) + C(2)*D (DDLE(-1)) + C(3)*D(DDLE(-2)) + C(4) *D(DDOM(-1)) + C(5)*D(DDOM(-2)) + C(6)*D(DOOPS(-1)) + C(7) *D(DOOPS(-2)) + C(8)*D(DPVTD(-1)) + C(9)*D(DPVTD(-2)) + C(10) *D(DGNIPC(-1)) + C(11)*D(DGNIPC(-2)) + C(12)*D(DEYS(-1)) + C(13)*D(DEYS(-2)) + C(14) As the coefficient is negative and the p-value is significant for the above model (shown in supporting information), it suggests a long-run association between DDLE to DDOM, DOOPS, DPVTD, DGNIPC, and DEYS. The result of vector error correction model shows that health care spending (current health expenditures, domestic health expenditures, out-of-pocket payments, and private health expenditures), anticipated years of schooling, and gross national income per capita have a long-run association with health outcomes i.e. life expectancy in South Asia.

### Granger causality test

Granger created a technique to examine the causal relationship between variables systematically [39]. In the Granger method, the causation from *y*_1,*t*_ to *y*_2,*t*_, we examine if the values of *y*_1,*t*_ in the past can contribute to an understanding of *y*_2,*t*_ in the present. We need to consider four instances for the bivariate model.

*Case 1*: *y*_1,*t*_
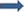
*y*_2,*t*_ *but y*_2,*t*_
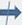
*y*_1,*t*_

*Case 2*: *y*_2,*t*_
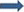
*y*_1,*t*_ *but y*_1,*t*_
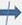
*y*_2,*t*_

*Case 3*: *y*_1,*t*_
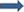
*y*_2,*t*_ *but y*_2,*t*_
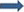
*y*_1,*t*_

*Case 4*: *y*_1,*t*_
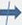
*y*_2,*t*_ *but y*_2,*t*_
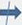
*y*_1,*t*_

Granger causality tests, which Granger first proposed in 1969, look at the pair-wise causal relationship between variables in a model that could result in a one-way, two-way, or no interaction [39]. The following equations can be used to represent the model when used to examine the causality between two variables (X and Y) at time t in time series:

$$X_{t}=\beta_{0}+\sum_{i=1}^{n}\beta_{1i}X_{t-i}+\sum_{i=1}^{n}\beta_{2i}Y_{t-i}+u_{1t}$$

$$Y_{t}=\beta_{3}+\sum_{i=1}^{n}\beta_{4i}Y_{t-i}+\sum_{i=1}^{n}\beta_{5i}X_{t-i}+u_{2t}$$

The relationship between various health care spending variables, anticipated years of education, gross national income per capita, and life expectancy in South Asian nations have been examined in this study. The pair-wise Granger causality test has been presented in Table 6 where, sample taken from 2000 to 2021with lag of 1.

**Table 6:** Pair-wise Granger causality test.

Null Hypothesis:	Obs.	F-Statistic	Prob.
DDOM does not Granger cause DDLE	135	0.03539	0.8511
DDLE does not Granger cause DDOM		0.58987	0.4438
DPVTD does not Granger cause DDLE	135	1.57040	0.2124
DDLE does not Granger cause DPVTD		1.56219	0.2136
DOOPS does not Granger cause DDLE	135	1.84227	0.1770
DDLE does not Granger cause DOOPS		1.61905	0.2055
DGNIPC does not Granger cause DDLE	152	0.23506	0.6285
DDLE does not Granger cause DGNIPC		0.42186	0.5170
DEYS does not Granger cause DDLE	152	0.00827	0.9277
DDLE does not Granger cause DEYS		1.22096	0.2710
DPVTD does not Granger cause DDOM	142	0.01549	0.9011
DDOM does not Granger cause DPVTD		4.43699	0.0370**
DOOPS does not Granger cause DDOM	142	0.19148	0.6624
DDOM does not Granger cause DOOPS		0.04341	0.8353
DGNIPC does not Granger cause DDOM	142	0.05138	0.8210
DDOM does not Granger cause DGNIPC		0.07416	0.7858
DEYS does not Granger cause DDOM	142	0.03446	0.8530
DDOM does not Granger cause DEYS		0.46006	0.4987
DOOPS does not Granger cause DPVTD	142	0.25929	0.6114
DPVTD does not Granger cause DOOPS		1.51928	0.2198
DGNIPC does not Granger cause DPVTD	142	1.74358	0.1889
DPVTD does not Granger cause DGNIPC		0.15842	0.6912
DEYS does not Granger cause DPVTD	142	4.61092	0.0335
DPVTD does not Granger cause DEYS		4.55717	0.0345
DGNIPC does not Granger cause DOOPS	142	2.46281	0.1188
DOOPS does not Granger cause DGNIPC		0.00691	0.9339
DEYS does not Granger cause DOOPS	142	1.88716	0.1717
DOOPS does not Granger cause DEYS		3.29290	0.0717
DEYS does not Granger cause DGNIPC	160	0.88230	0.3490
DGNIPC does not Granger cause DEYS		1.05307	0.3064

Table 6 shows no short-run causality between health care spending and other variables. However, there is unidirectional causality from DDOM to DPVTD and bidirectional causality between DEYS and DPVTD. The pair-wise Granger causality test indicates that health care spending (current health expenditures, domestic health expenditures, out-of-pocket payments, and private health expenditures), anticipated years of schooling, and gross national income per capita do not have a short-run association with health outcomes i.e. life expectancy in South Asia.

### The Wald test statistics

Furthermore, Wald test statistics were used to calculate the short-run combined effects of the independent variables on the dependent variable. Accepting the null hypothesis implies that there is no short-run causality exists between the dependent and independent variables [38]. The following null hypothesis has been proposed for a short-term relationship.

Null Hypothesis: C(4) = C(5) = C(6) = C(7) = C(8) = C(9) = C(10) = C(11) = C(12) = C(13)

Table 7 presents the results of the Wald test statistics.

**Table 7:** Wald test statistic.

Test Statistic	Value	df	Probability
Chi-square	16.11075	9	0.0646

Table 7 suggests that there is no short-run causality between DDLE and other independent variables, as the null hypothesis is rejected. The Wald test statistics do not find short-run causality between life expectancy and health care spending indicators including gross national income per capita, and anticipated years of schooling in South Asia.

### Diagnostic test of residuals

For further validation of the model, the residual should be normally distributed and have homoscedasticity and no serial correlation. This assumption of the regression model has been presented below.

### Normality test

**Fig 1:**
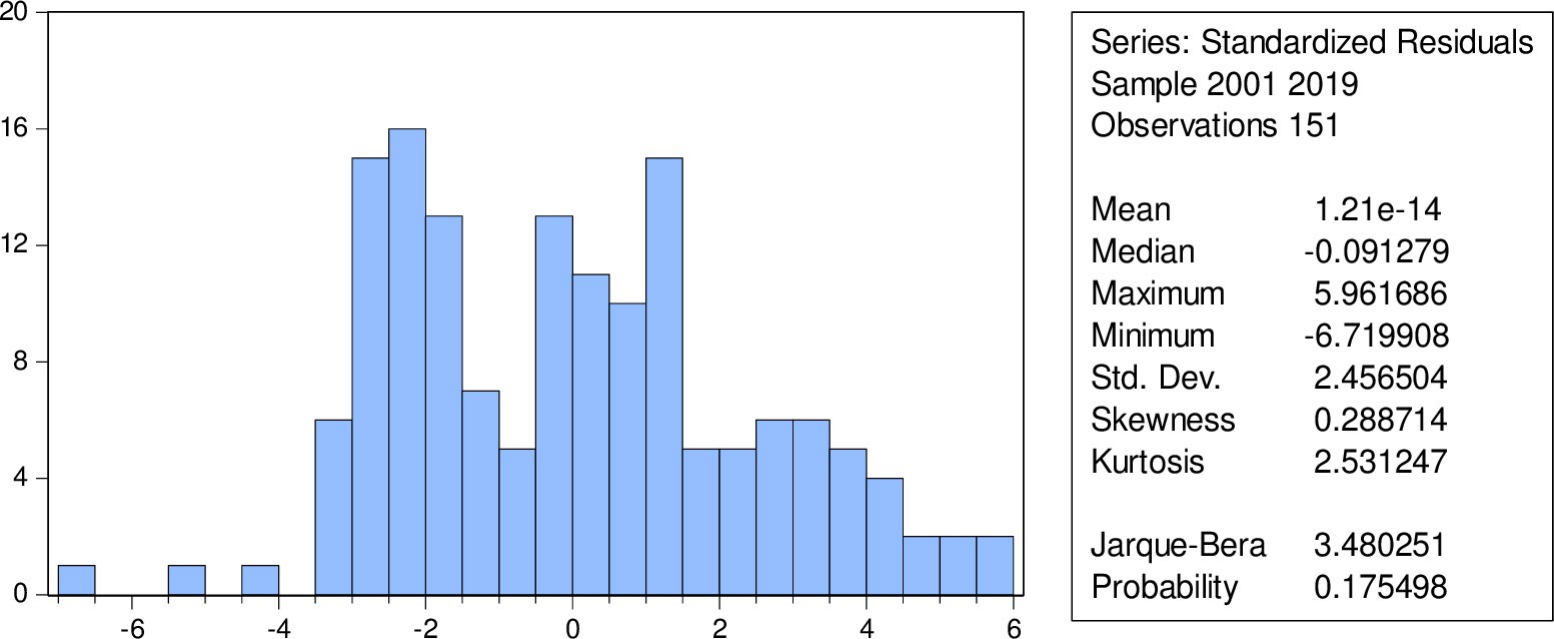
Normality test.

Normality test was performed to identify whether or not the model’s residual was normally distributed. If the residuals are not normally distributed, the model’s results violate the assumption of the central limit theorem, rendering regression results invalid. The Jarque-Bera test was performed to identify the normality of the model [40]. The Jarque-Bera statistics are 3.4803, with a p-value of 0.1755, indicating that we failed to reject the null hypothesis. It implies that residuals are normally distributed, which validates the model’s assumption (Fig 1).

Breush Pegan test was performed to validate the assumption of homoscedasticy. To improve the model’s validity, the residual should be homoscedastic. If the probability value exceeds 5%, we have strong evidence that the residuals are homoscedastic [41]. The p-value in Table 8 is greater than 5%, so we can accept the null hypothesis. It suggests that the residuals are homoscedastic, which validates the model’s assumption.

**Table 8:** Breusch-Pagan test.

Test	Statistic	d.f.	Prob.
Breusch-Pagan LM	34.15925	28	0.1957
Pesaran scaled LM	0.823064		0.4105
Pesaran CD	1.427649		0.1534

The study examines the life expectancy and health care spending, anticipated years of schooling, and gross national income per capita of South Asian countries. The econometric model results indicate that health care spending, anticipated years of schooling, and gross national income per capita all have a long-run relationship with health outcomes i.e. life expectancy. However, pair-wise Granger causality and Wald test statistics reveal no short-run causality between life expectancy and health care spending indicators including gross national income per capita, and anticipated years of schooling in South Asia.

Previous study has demonstrated that total health spending has considerable impact on average life expectancy [42,43]. Improvements in infant mortality correlate with increased health care spending, but they are also linked to life expectancy [44]. Public and private health care spending improves the health outcomes significantly [45–47]. The average number of school years and access to sanitary facilities are both strong predictors of life expectancy [48]. The findings of this study highlight the importance greater government spending on health care in enhancing access to and quality of health services. However, health coverage in many South Asian countries is primarily based on out-of-pocket costs, posing significant challenges for individuals and families. High out-of-pocket costs, as well as insufficient public funds and limited access to health insurance, frequently leads to budget constraints, and reduces essential health services [49].

The causal relationship between education and health is significant. The outcome of this study is consistent with the other researchers who emphasized the importance of income and education in improving health outcomes [50,51]. Health and its components, such as harmful environments, risky behaviours, and the use of preventative services, are strongly correlated with education [52,53]. Individuals with a high level of education are more likely to adopt healthy behaviours and make informed health decisions [54]. Average life expectancy and enrolment in education are directly correlated [55]. People with more years of schooling tend to have better health, more well-being, and healthier habits [56–58]. This is especially significantin the South Asian context, where disparities in educational attainment and health outcomes are evident.

There is a long-term relationship between health outcomes and per capita gross national income and health care spending [59,60]. The growth in health spending and GDP across income levels have a different causal relationship When cross-sectional dependence in the panel is taken into account, the causal link between increase in health spending and GDP across income level changes, and the results demonstrate no long-term cointegration [61].

The growing rate of per capita income has greatly increased life expectancy [4]. When compared to private health spending, public health expenditure considerably extends life expectancy [62]. Spending on health care can enhance life quality and health outcomes while lowering financial obstacles to accessing care [63].

## Conclusions

This study finds a long-run correlation between health care expenditure and health outcomes in the South Asian region. Life expectancy is strongly impacted byhealth care spending, gross national income per capita, and anticipated years of schooling. It is vital to consider an efficacious health strategy that may improve and promote individuals’ healthy life expectancy and overall welfare. Enhancing education and increasing student enrolment need to be essential components of health care.

The government may enhance school enrolment to increase health literacy and health outcomes in South Asia. As a result, persons with more years of schooling have better health, higher levels of well-being, healthier habits, and, ultimately, a longer life expectancy. This study will provide an important insight to policymakers on improving health outcomes through targeted and sustained health care spending in South Asia.

This study is confined to assess health care spending (current health expenditures, domestic health expenditures, out-of-pocket payments, and private health expenditures), GNIPC, and EYS with health outcomes as a life expectancy in South Asian nations using econometric analysis. This study only has access to secondary data from World Bank Statistics from 2000 to 2021, but in some situations data are available up to 2019. There are no biases in the selection of data and variables for this study. Further study can be conducted by comparing with East Asia and other similar settings.

## Supporting information

S1 Fig Model validity and approval. (DOCX)

S1 File Links and process of data extraction. (DOCX)

## References

[1] Briggs AM, Shiffman J, Shawar YR, Åkesson K, Ali N, Woolf AD. Global health policy in the 21st century: Challenges and opportunities to arrest the global disability burden from musculoskeletal health conditions. Best Practice & Research Clinical Rheumatology. 2020;34(5):101549. doi: 10.1016/j.berh.2020.101549 32713802 PMC7377715

[2] Fullman N, Yearwood J, Abay SM, Abbafati C, Abd-Allah F, Abdela J, et al. Measuring performance on the Healthcare Access and Quality Index for 195 countries and territories and selected subnational locations: A systematic analysis from the Global Burden of Disease Study 2016. The Lancet. 2018;391(10136):2236–2271. doi: 10.1016/S0140-6736(18)30994-2 29893224 PMC5986687

[3] Gallet CA, Doucouliagos H. The impact of healthcare spending on health outcomes: A meta-regression analysis. Social Science & Medicine. 2017;179:9–17. doi: 10.1016/j.socscimed.2017.02.024 28237460

[4] Rahman MM, Khanam R, Rahman M. Health care expenditure and health outcome nexus: New evidence from the SAARC-ASEAN region. Globalization and Health. 2018;14(1): 113. doi: 10.1186/s12992-018-0430-1 30466452 PMC6249744

[5] Bradley EH, Elkins BR, Herrin J, Elbel B. Health and social services expenditures: Associations with health outcomes. BMJ Quality & Safety. 2011;20(10):826–831. doi: 10.1136/bmjqs.2010.048363 21447501

[6] Stiefel MC, Perla RJ, Zell BL. A healthy bottom line: Healthy life expectancy as an outcome measure for health improvement efforts. The Milbank Quarterly. 2010; 88(1): 30–53. doi: 10.1111/j.1468-0009.2010.00588.x 20377757 PMC2888015

[7] Zaidi S, Saligram P, Ahmed S, Sonderp E, Sheikh K. Expanding access to healthcare in South Asia. BMJ. 2017;11;357. doi: 10.1136/bmj.j1645 28400377

[8] Razvi S, Khan AU. Health financing in South Asia—The role of public–private partnerships. South Asian Survey. 2015;22(1):15–36. 10.1177/0971523117695143.

[9] Yip W. Healthcare system challenges in Asia. In Oxford Research Encyclopedia of Economics and Finance 2019 May 23. 10.1093/acrefore/9780190625979.013.245.

[10] Haque M, Islam T, Rahman NA, McKimm J, Abdullah A, Dhingra S. Strengthening primary health-care services to help prevent and control long-term (chronic) non-communicable diseases in low-and middle-income countries. Risk management and healthcare policy. 2020 May 18:409–26. 10.2147/RMHP.S239074. PMC7244358 32547272

[11] European Commission. Healthy life years. 2009; Available at http://ec.europa.eu/health/ ph_information/in.

[12] Wilkie J, Young A. Why health matters for economic performance. Economic Round-Up. 2009;1:57–72. 10.3316/ielapa.745349836827886.

[13] Sepehri A. A critique of Grossman’s canonical model of health capital. International Journal of Health Services. 2015;45(4):762–78. doi: 10.1177/0020731415586407 25995307

[14] Grossman M. On the concept of health capital and the demand for health. In determinants of health: an economic perspective. Columbia University Press. 2017.

[15] Wagstaff A. The demand for health: theory and applications. Journal of Epidemiology & Community Health. 1986 Mar 1;40(1):1–1. doi: 10.1136/jech.40.1.1 3711765 PMC1052481

[16] Preston SH. The changing relation between mortality and level of economic development. Population Studies. 1975;29(2):231–48. 10.1080/00324728.1975.10410201. 11630494

[17] Becker GS. Human capital: A theoretical and empirical analysis, with special reference to education. University of Chicago press; 2009.

[18] Nixon J, Ulmann P. The relationship between health care expenditure and health outcomes. The European Journal of Health Economics. 2006;7(1):7–18. 10.1007/s10198-005-0336-8. 16429295

[19] Chireshe J, Ocran MK. Health care expenditure and health outcomes in sub‐Saharan African countries. African Development Review. 2020 Sep;32(3):349–61. 10.1111/1467-8268.12444.

[20] Rana RH, Alam K, Gow J. Health expenditure and gross domestic product: Causality analysis by income level. International Journal of Health Economics and Management. 2020;20(1):55–77. doi: 10.1007/s10754-019-09270-1 31313127

[21] Halıcı-Tülüce NS, Doğan İ, Dumrul C. Is income relevant for health expenditure and economic growth nexus? International Journal of Health Economics and Management. 2016;16(1):23–49. doi: 10.1007/s10754-015-9179-8 27878709

[22] Stuckler D, Basu S, Suhrcke M, Coutts A, McKee M. The public health effect of economic crises and alternative policy responses in Europe: an empirical analysis. The Lancet. 2009 Jul 25;374(9686):315–23. doi: 10.1016/S0140-6736(09)61124-7 19589588

[23] Grigorakis N, Galyfianakis G, Tsoukatos E. Assessing the responsiveness of out-of-pocket healthcare expenditure to macro-fiscal factors and different health financing systems: evidence from the European and OECD area. EuroMed Journal of Business. 2022 May 17;17(2):193–217. 10.1108/EMJB-09-2020-0105.

[24] Berkman ND, Sheridan SL, Donahue KE, Halpern DJ, Crotty K. Low health literacy and health outcomes: An updated systematic review. Annals of Internal Medicine. 2011;155(2): 97–107. doi: 10.7326/0003-4819-155-2-201107190-00005 21768583

[25] DeWalt DA, Berkman ND, Sheridan S, Lohr KN, Pignone MP. Literacy and health outcomes: A systematic review of the literature. Journal of general internal medicine. 2004 Dec;19:1228–39. doi: 10.1111/j.1525-1497.2004.40153.x 15610334 PMC1492599

[26] De Neve JE, Sachs JD. Sustainable development and human well-being. World happiness report. 2020;112–127.

[27] Qaiser Gillani D, Gillani SAS, Naeem MZ, Spulbar C, Coker-Farrell E, Ejaz A, et al. The nexus between sustainable economic development and government health expenditure in Asian countries based on ecological footprint consumption. Sustainability. 2021;13(12): 6824.

[28] Jadhav S, Yeravdekar R, Kulkarni M. Cross-border healthcare access in South Asian Countries: Learnings for sustainable healthcare tourism in India. Procedia-Social and Behavioral Sciences. 2014;157:109–117.

[29] Mukherjee M, Abhinay K, Rahman MM, Yangdhen S, Sen S, Adhikari BR, et al. Extent and evaluation of critical infrastructure, the status of resilience, and its future dimensions in South Asia. Progress in Disaster Science. 2023;17:100275.

[30] The World Bank (WB). The World Bank Statistics. https://data.worldbank.org/indicator.

[31] World Health Organization (WHO). Global Health Expenditure Database. https://apps.who.int/nha/database/Select/Indicators/en.

[32] National Research Council. Defining and Measuring Population Health. In: Council NR, editor. Accounting for health and health care: approaches to measuring the sources and costs of their improvement. Washington (DC): National Academies Press (US); 2011.

[33] Bell A, Jones K. Explaining fixed effects: Random effects modeling of time-series cross-sectional and panel data. Political Science Research and Methods. 2015 Jan;3(1):133–53. 10.1017/psrm.2014.7 P.

[34] Elhorst JP, Gross M, Tereanu E. Cross‐sectional dependence and spillovers in space and time: Where spatial econometrics and global VAR models meet. Journal of economic surveys. 2021 Feb;35(1):192–226. 10.1111/joes.12391.

[35] Leybourne SJ, McCabe BPM, Tremayne AR. Can economic time series be differenced to stationarity? Journal of Business & Economic Statistics. 1996;14(4):435–446. 10.1080/07350015.1996.10524673.

[36] Hong Y, Wang X, Wang S. Testing strict stationarity with applications to macroeconomic time series. International Economic Review. 2017;58(4):1227–1277. 10.1111/iere.12250.

[37] MaCurdy TE. The use of time series processes to model the error structure of earnings in longitudinal data analysis. Journal of Econometrics. 1982;18(1):83–114. 10.1016/0304-4076(82)90096-3.

[38] Granger CWJ. Investigating causal relations by econometric models and cross-spectral methods. Econometrica. 1969;37(3):424–438. doi: 10.2307/1912791

[39] Johansen S, Juselius K. Maximum likelihood estimation and inference on cointegration—With appucations to the demand for money. Oxford Bulletin of Economics and Statistics. 1990; 52(2):169–210.

[40] Thadewald T, Büning H. Jarque–Bera test and its competitors for testing normality–A power comparison. Journal of Applied Statistics. 2007;34(1):87–105. 10.1080/02664760600994539.

[41] Đalić I, Terzić S. Violation of the assumption of homoscedasticity and detection of heteroscedasticity. Decision Making: Applications in Management and Engineering. 2021;4(1): Article 1. 10.31181/dmame2104001d.

[42] Radmehr M, Adebayo TS. Does health expenditure matter for life expectancy in Mediterranean countries? Environmental Science and Pollution Research. 2022;29(40): 60314–60326. doi: 10.1007/s11356-022-19992-4 35420335 PMC9008298

[43] Bein MA, Unlucan D, Olowu G, Kalifa W. Healthcare spending and health outcomes: Evidence from selected East African countries. African Health Sciences. 2017;17(1): Article 1. doi: 10.4314/ahs.v17i1.30 29026399 PMC5636241

[44] Tanaka T, Okamoto S, Canning D. National health spending, healthcare resources, service utilization, and health outcomes. American Journal of Epidemiology. 2022;191(3): 386–396. doi: 10.1093/aje/kwab179 34128527

[45] Nketiah-Amponsah E. The impact of health expenditures on health outcomes in Sub-Saharan Africa. Journal of Developing Societies. 2019;35(1):134–152. 10.1177/0169796X19826759.

[46] Akinci F, Hamidi S, Suvankulov F, Akhmedjonov A. Examining the impact of health care expenditures on health outcomes in the Middle East and N. Africa. Journal of Health Care Finance. 2014;41(1). http://www.healthfinancejournal.com/~junland/index.php/johcf/ article/view/6.

[47] Farag M, Nandakumar AK, Wallack S, Hodgkin D, Gaumer G, Erbil C. Health expenditures, health outcomes and the role of good governance. International Journal of Health Care Finance and Economics. 2013;13(1):33–52. doi: 10.1007/s10754-012-9120-3 23266896

[48] Jafrin N, Masud MM, Seif ANM, Mahi M, Khanam M. A panel data estimation of the determinants of life expectancy in selected SAARC countries. Operations Research and Decisions. 2021;31(4):69–87.

[49] World Health Organization. New perspectives on global health spending for universal health coverage. World Health Organization; 2017.

[50] Anand S, Ravallion M. Human development in poor countries: on the role of private incomes and public services. Journal of economic perspectives. 1993 Feb 1;7(1):133–50.

[51] Lindberg MH, Chen G, Olsen JA, Abelsen B. Combining education and income into a socioeconomic position score for use in studies of health inequalities. BMC public health. 2022 May 1 3;22(1):969. doi: 10.1186/s12889-022-13366-8 35562797 PMC9107133

[52] Adams RJ. Improving health outcomes with better patient understanding and education. Risk Management and Healthcare Policy. 2010;3:61–72. doi: 10.2147/RMHP.S7500 22312219 PMC3270921

[53] Feinstein L, Sabates R, Anderson TM, Sorhaindo A, Hammond C. What are the effects of education on health? Measuring the Effects of Education on Health and Civic Engagement: Proceedings of the Copenhagen Symposium. 2006;171–354.

[54] Grossman M. Education and nonmarket outcomes. Handbook of the Economics of Education. 2006 Jan 1;1:577–633.

[55] Bunyaminu A, Mohammed I, Yakubu IN, Shani B, Abukari AL. The effect of health expenditure on average life expectancy: Does government effectiveness play a moderating role? International Journal of Health Governance. 2022;27(4):365–377. 10.1108/IJHG-03-2022-0027.

[56] Cutler DM, Lleras-Muney A. Education and health: Insights from international comparisons. 2012;(Working Paper No. 17738). National Bureau of Economic Research. 10.3386/w17738.

[57] Spasojević J. Effects of education on adult health in Sweden: Results from a natural experiment. In Slottje D. & Tchernis R.(Eds.). Current Issues in Health Economics. 2010; 290:179–199). 10.1108/S0573-8555(2010)0000290012.

[58] Lee RLT, Loke AJTY. Health-promoting behaviours and psychosocial well-being of university students in Hong Kong. Public Health Nursing. 2005;22(3):209–220. 10.1111/j.0737-1209.2005.220304.x. 15982194

[59] Momoh OA, Okwu AT. Income and health outcomes in the anglophone West African countries: A dynamic heterogeneous approach. Journal of Economics and Allied Research. 2022;7(1): Article 1.

[60] Hermanowski T, Bystrov V, Staszewska-Bystrova A, Orlewska E. Analysis of trends in life expectancies and per capita gross domestic product as well as pharmaceutical and non-pharmaceutical healthcare expenditures. Acta Pol Pharm. 2015; 2(5):1045–1050. 26665412

[61] Rodríguez AF, Nieves Valdés M. Health care expenditures and GDP in Latin American and OECD countries: a comparison using a panel cointegration approach. International Journal of Health Economics and Management. 2019 Jun 15;19(2):115–53. doi: 10.1007/s10754-018-9250-3 30267372

[62] Jelani QU, Jhamnani S, Spatz ES, Spertus J, Smolderen KG, Wang J, et al. Financial barriers in accessing medical care for peripheral artery disease are associated with delay of presentation and adverse health status outcomes in the United States. Vascular Medicine. 2020 Feb;25(1):13–24. doi: 10.1177/1358863X19872542 31603393

[63] Singh S, Bala MM, Kumar N. The dynamics of public and private health expenditure on health outcome in Southeast Asia. Health & Social Care in the Community. 2022 Sep;30(5):e2549–58. doi: 10.1111/hsc.13698 34981612

