# Supplementary material for: Life expectancy and health care spending in South Asia: An econometric analysis
Source: PLoS One. 2024 Dec 23;19(12):e0310153. doi: 10.1371/journal.pone.0310153 (PMC11666019; doi:10.1371/journal.pone.0310153)
Supplement: S1 Fig — (DOCX) [file pone.0310153.s001.docx]

**Supplementary file-1:**

**Model validity and approval**

Used software:

R/ RStudio

Packages/libraries used:

tidyverse, car, metan

Checking for autocorrelation among the independent variables


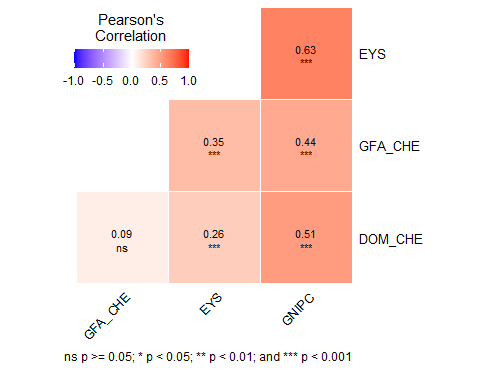


Used package for correlation: metan

Checking the normality using histogram


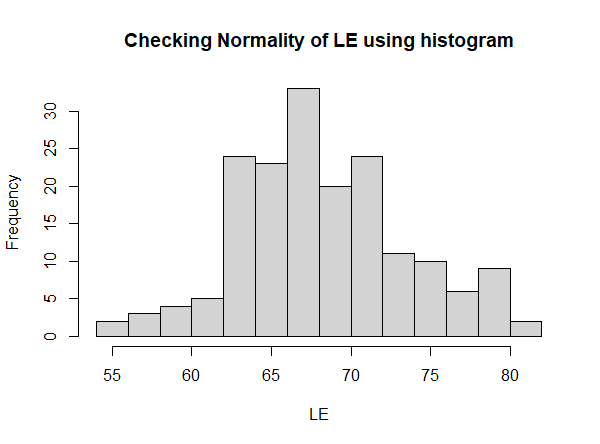


Assumptions Checking


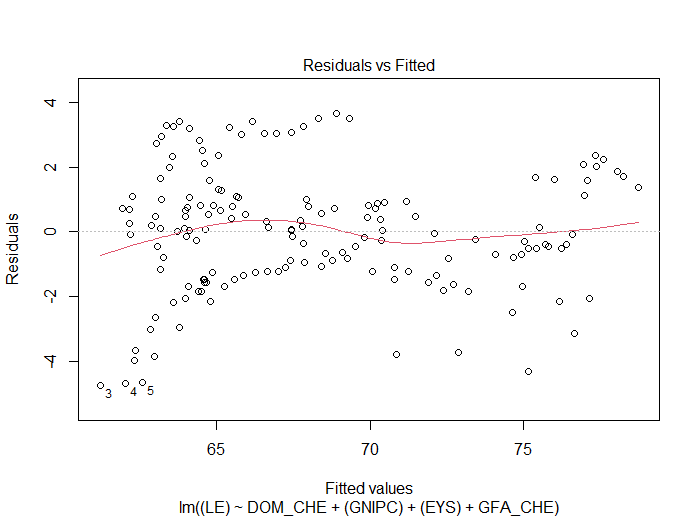


Normal Q-Q Plot


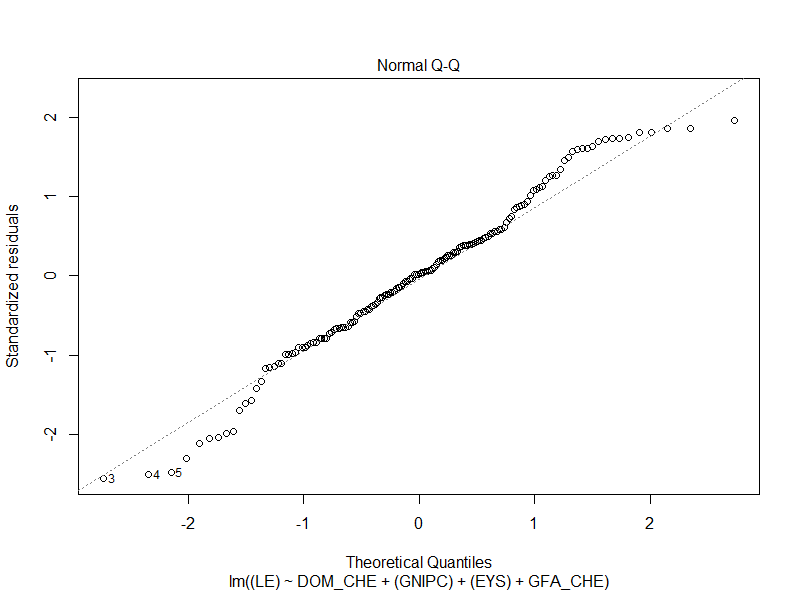


Residuals vs Leverage


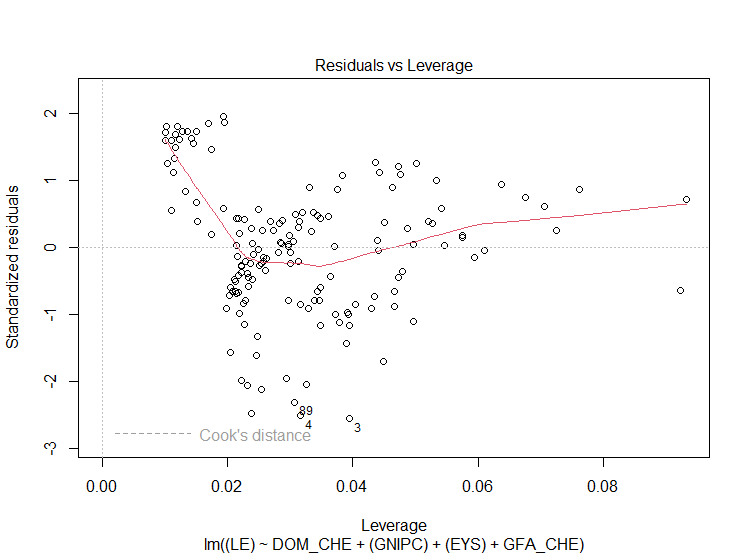


VIF CHECKING:

Library used: car

Vif of model:

DOM_CHE GNIPC EYS GFA_ CHE

1.412595 2.415823 1.694857 1.289563
