# Supplementary material for: Life expectancy and health care spending in South Asia: An econometric analysis
Source: PLoS One. 2024 Dec 23;19(12):e0310153. doi: 10.1371/journal.pone.0310153 (PMC11666019; doi:10.1371/journal.pone.0310153)
Supplement: S1 File — (DOCX) [file pone.0310153.s002.docx]

**Links and process of data extraction**

**Data Bank: World Bank Group**

Link: [https://databank.worldbank.org/reports.aspx?source=2&series=SH.XPD.CHEX.GD.ZS&country=#](https://databank.worldbank.org/reports.aspx?source=2&series=SH.XPD.CHEX.GD.ZS&country=)

**Process of data extraction:** Based on the above links, we followed the following steps to extract the data.

1. **Database selection:** Selected World Development Indicators
2. **Country selection:** Selected Afghanistan, Bangladesh, Bhutan, India, Maldives, Nepal, Pakistan, and Sri Lanka
3. **Series selection:** Selected Current health expenditures (% of GDP)
4. **Time selection:** Selected the years from 2000 to 2023

Based on the above links and above process, we collected the data for all variables: Life expectancy (LE), Domestic health expenditures (DOM), Out-of-pocket payments (OOPS), Private health expenditures (PVTD), Expected years of schooling (EYS), and Gross national income per capita (GNIPC).
